# New panel of biomarkers to discriminate between amelanotic and melanotic metastatic melanoma

**DOI:** 10.3389/fonc.2022.1061832

**Published:** 2023-01-26

**Authors:** Ioana V. Militaru, Alina Adriana Rus, Cristian V.A. Munteanu, Georgiana Manica, Stefana M. Petrescu

**Affiliations:** ^1^ Department of Molecular Cell Biology, Institute of Biochemistry, Bucharest, Romania; ^2^ Department of Bioinformatics and Structural Biochemistry, Institute of Biochemistry, Bucharest, Romania

**Keywords:** melanoma biomarkers, amelanotic melanoma, mass spectrometry, proteomics, melanoma diagnostic, melanoma prognostic

## Abstract

Melanoma is a form of skin cancer that can rapidly invade distant organs. A distinctive feature of melanomas is their pigmentation status, as melanin is present in most skin melanomas, whilst many metastatic tumors could become amelanotic. Besides the obvious malfunction of the key genes of the melanin pathway, the amelanotic tumors could bear a characteristic molecular signature accounting for their aggressivity. Using mass spectrometry-based proteomics we report here a distinctive panel of biomarkers for amelanotic aggressive melanoma that differ from the less invasive pigmented cells. The developed method allows the label-free quantification of proteins identified by LC-MS/MS analysis. We found a set of proteins comprising AHNAK, MYOF, ANXA1, CAPN2, ASPH, EPHA2, THBS1, TGM2, ACTN4 along with proteins involved in cell adhesion/migration (integrins, PLEC, FSCN1, FN1) that are highly expressed in amelanotic melanoma. Accompanying the down regulation of pigmentation specific proteins such as tyrosinase and TYRP1, these biomarkers are highly specific for a type of highly invasive melanoma. Interestingly, the LC-MS/MS proteomics analysis in hypoxia revealed that the abundance of this specific set of proteins found in normoxia was rather unaltered in these conditions. These biomarkers could therefore predict a metastatic behaviour for the amelanotic cells in the early stages of the tumor development and thus serve in melanoma prognostic. Applying this algorithm to related databases including melanoma samples published by independent laboratories/public databases we confirm the specificity of the newly found signatures. Overall, we begin to unravel the molecular alterations in the amelanotic melanoma and how basic proteomics offers insights into how to assess the clinical, pathological and misdiagnosis differences between the main subtypes of melanoma.

## 1 Introduction

Cutaneous melanoma is an aggressive type of skin cancer, counting tens of thousands of deaths each year. Its increased metastatic potential is supported by genetic mutations and microenvironmental alterations within the tumor, making this type of cancer one of the most deadliest (1).

A particularity of these cells is defined by their melanin synthesis capacity. This process takes place in melanosomes, where the melanin precursor, tyrosine, undergoes hydroxylation and subsequent oxidation reactions. Melanin synthesis is dependent on the activity of melanogenesis enzymes, of which tyrosinase (TYR) is indispensable for initiating the process (2). Besides being a diagnosis tool, pigmentation also impacts cells behaviour to a large extent, by conferring them distinctive features (3). A small percentage of encountered melanoma cases is assigned to amelanotic melanoma. Amelanotic lesions are from the beginning difficult to recognize and as time goes by, they are prone to be more advanced at the time of diagnosis with a considerable decrease in the overall survival (4). They often present some aggressive features and rapid tumor growth (5). Proteins related to cell migration and invasion deserve a closer attention for a better clinical outcome assessment and for an optimal treatment strategy (6, 7). There are some important delays in the spreading and metastasis of pigmented as opposed to amelanotic cancer cells, mainly due to cell elasticity impairment (8). Furthermore, some proteins are thought to play a central role in cell spreading process. The chondroitin sulfate proteoglycan 4 (9), carcinoembryonic antigen-related cell adhesion molecule (10), galectin-3 (11) and several matrix metalloproteinases (12) are some of the proteins involved in melanoma progression and metastasis. There is a need of continuous and overall evaluation of proteomic profiling of melanoma cell lines. Several biomarkers were proposed for melanoma subtype differentiation. Vimentin, nestin, annexin A1 (ANXA1) and fibronectin (FN1) were reported as predictive biomarkers for aggressive melanoma (13). Furthermore, comparative analysis between melanocytes and melanoma cells demonstrated that hepatoma-derived growth factor and nucleophosmin B23 present increased expression in malignant cell lines (14). Another approach in discovering new biomarkers related to metastatic potential was based on differences between highly metastatic and low metastatic melanoma cells (15). All these comparative approaches may help to better understand the molecular mechanisms underlying melanoma progression and metastasis.

Pigmented melanomas can be recognized due to their high melanin content, as opposed to amelanotic melanomas that lack pigmentation. As a result, amelanotic melanoma is more likely to be misdiagnosed as basal carcinoma, nevus or seborrheic keratosis and is usually associated with poor patient outcome. These elements underscore the need of a more complex set of biomarkers for an early and accurate diagnosis. We found mass spectrometry as a promising approach for the characterization of different melanoma cell lines by offering the possibility of massive analysis of proteins amounts within the cells. Our purpose was to connect cell migration capacity to certain migration related proteins, which could provide valuable information about tumor progression and overall survival rate.

In this regard, we used mass-spectrometry based proteomics for a comparative proteome evaluation between pigmented and non-pigmented melanoma cell lines. Proteomic data was supported by cell migration assays in order to appreciate cell ability to spread and several candidate biomarkers were selected. The cellular response to hypoxia was also monitored in order to evaluate the alterations ocurring at the proteome level during the oxygen deprivation, a condition often encountered during tumour growth. We propose a panel of biomarkers whose altered expression could be a characteristic of the amelanotic melanoma and may lead to a better understanding of the pigmentation related processes and serve as new diagnostic tool in the evaluation criteria of amelanotic melanoma.

## 2 Materials and methods

*Cell lines and antibodies*: HEK293T, A375, SKMEL28, SKMEL23 and MNT1 cells were from the European Collection of Animal Cell Cultures. LAU-ME290 (Me290) is a gift from Professor Romero from Ludwig Institute for Cancer Research, Lausanne Branch, Switzerland (16). Anti-HIF1α (AF1935) and anti-HIF2α (AF2886) antibodies were from R&D Systems. Anti-p44/42 MAP Kinase (ERK1/2) (4695), anti-Phospho-p44/42 MAPK (pErk1/2) (4377) and anti- β-Catenin (D10A8) antibodies were from Cell Signaling Technology. Anti-MITF (ab80651) antibody, anti-SQSTM1/p62 antibody (ab155686), anti-calnexin antibodies (ab22595) were from Abcam. Anti-AHNAK (sc-390743), anti-cathepsin D (sc-377299), anti-Tyrosinase (sc-20035), anti-ITGA3 (sc-374242), anti-Pmel17 (sc393094), anti-TRP1 (sc166857), anti-TRP2/DCT (sc-74439) antibodies were from Santa Cruz Biotechnology, anti-LC3 antibody (LC3-2G6) was from NanoTools and anti-fibronectin antibody (F3648) was from Sigma.

*Cell culture*: HEK293T and A375 cells were cultured in DMEM (cat: 31966-021, Gibco) supplemented with 10% heat-inactivated fetal bovine serum (cat: 10270-098, Gibco). MNT1 cells were cultured in DMEM (cat: 31966-021, Gibco) supplemented with 20% heat-inactivated fetal bovine serum (cat: 10270-098, Gibco). SKMEL28, SKMEL23 and Me290 cells were cultured in RPMI 1640 (cat: 61870-10, Gibco) supplemented with 10% heat-inactivated fetal bovine serum (cat: 10270-098, Gibco). Cells were cultured under standard culture conditions (37°C with 20% O2, 5% CO2). Hypoxic experiments were performed in a hypoxic chamber (InvivO_2_ 300 hypoxic chamber, Baker Ruskinn) in the presence of 1% O2 and 5% CO2 for 24 hours.

*Migration assay*: Migration assay was performed using dextran-PEG hydrogel SG (Cellendes GmbH) for setting the migration area limits. 1.5 μl gel was placed in each well of a 96 well plate. 30 minutes later cells were plated around the area covered by the gel. Depending on the cell line, the cells were seeded as follows: 10^4^ cells of A375 and SKMEL28, 2x10^4^ cells of MNT1 and 1.5x10^4^ cells of Me290 and SKMEL23. After 24 hours, the gel was dissolved using dextranase (3-D Life Dextranase Cellendes GmbH) (1:20 in cell media). Cells were washed one time with PBS and incubated with the corresponding media. Representative images of migrating cells were taken after 2 days. Image acquisition was performed by using Tissue FAXS PLATES software module. Image J software was used to quantify the percentage of area coverage. Statistical analysis was performed using GraphPad Prism 9 software.

*Western blot*: Cells were lysed in RIPA buffer (10 mM Tris-Cl pH 8, 1 mM EDTA, 0.5 mM EGTA, 1% NP40, 0.1% sodium deoxycholate, 0.5% SDS,140 mM NaCl). Cell lysates were separated by 10% SDS-PAGE and transferred on nitrocellulose or polyvinylidene difluoride (PVDF) membranes. The membranes were probed with the appropriate primary and secondary antibodies. Image J software was used in order to quantify the corresponding bands. Statistical analysis was performed using GraphPad Prism 9 software.

*Sample preparation for Mass Spectrometry*: Cells were lysed in a buffer containing 6M guanidine-HCl in 100 mM Tris-HCl pH 8.5 and subsequently sonicated and centrifuged at 14000 x g for 30 minutes at room temperature. Proteins were quantified using bicinchoninic acid (BCA) assay and a second quantification was made before injection by measuring the protein absorbance at NanoDrop at 280 nm. The disulfide bonds were further reduced with 10 mM dithiothreitol (DTT) and incubated for 45 minutes at 56°C. Cysteine sites were alkylated with 55mM iodoacetamide for 45 minutes, in the dark. The alkylating agent was neutralized by adding an equivalent amount of DTT. Endo-LysC enzyme mixture was added in a ratio of 1:100 (protease: protein) and samples were incubated for 4 hours at 37°C. After buffer dilution to less than 1M guanidine concentration, the proteins were subjected to trypsin proteolysis. The trypsin: protein ratio was 1:100. The samples were incubated at 37°C overnight and the digestion was stopped with formic acid (FA). The salts were removed using C18 Pierce™ C18 Tips, 100 µL bed (Cat No.: 87784) as described in the protocol provided by the manufacturer. The eluted peptides were further concentrated in a Speed-Vac.

*Immunofluorescence assay:* Immunofluorescence was performed as previously described (17). Briefly, cells were seeded on coverslips into 12 wells plate (3x10^4^ cells per well), fixed in 1% paraformaldehyde, permeabilized with 0.1% Triton X-100 and subsequently blocked with 1% BSA. Cells were subjected to immunofluorescence staining with anti-AHNAK antibody (1:400) for 1 hour, at room temperature. Further, cells were incubated with Alexa 488-labeled anti-mouse secondary antibody (1:1000) and examined using a Zeiss-LSM confocal microscope.

*LC-MS/MS analysis*: Before injection peptides were reconstituted in solvent A (0.06% FA, 2% acetonitrile - ACN) and injected for nanoLC-MS/MS analysis using an Easy-nanoLC II (Thermo Fisher Scientific) connected online to an LTQ-Orbitrap Velos Pro instrument (Thermo Fisher Scientific). Peptides were first loaded on an Acclaim™ PepMap™ 100 C18 HPLC trap column (Thermo Fisher Scientific Cat No 164199, 5 μm, 0.1 mm x 20 mm) and then separated with a flow rate of 300 nL/min on a Acclaim™ PepMap™ C18 Reversed Phase HPLC Column (Thermo Fisher Scientific Cat No 160321, 3 μm, 0.075 mm x 150 mm) and detected as previously described (18, 19). Briefly, a 240 min 2-30% solvent B (0.06% FA, 80% ACN) gradient was used and the eluted peptides were detected in the orbital trap at 60 000 resolution (m/z 400). The survey scan was followed by MS/MS fragmentation of top 15 most abundant ions and the detection of the fragment ions in the linear trap. Dynamic exclusion was activated with a repeat count of 1exclusion duration of 60 s and a list size of 500. To avoid redundant fragmentation exclusion lists were used between technical replicates.

*Data analysis*: Raw data were searched against the human version of the UniProtKB database, using the Andromeda algorithm integrated into the MaxQuant environment (20) with Trypsin/P as the selected protease using maximum 2 missed cleavages, Cys carbamidomethylation as a fixed modification and Met oxidation and protein N-terminus acetylation as variable modifications. The following settings were used during the searches: 20 ppm maximum allowed mass deviation for the first search and 4.5 ppm for the main search for ions detected in the Orbitrap and 0.5 Da for fragment ions detected in the linear trap. The results were filtered at 1% FDR at PSM and protein level with the MaxQuant default algorithm.

Proteomic data analysis has been conducted in Perseus software (21), version 2.0.6.0. Protein abundance quantification was made considering the LFQ intensity. Three biological replicates for each condition were used in order to perform statistical testing. Proteomic data was filtered first for identifications from the reversed database and proteins identified by site. The LFQ values were log2 transformed. Protein quantitation method required a minimum of 2 unique peptides per protein and at least three valid values in at least one group defined as a cell line. Missing values were imputed by values from a normal distribution (width = 0.5, shift = 1.8 for all cell lines, except Me290 and SKMEL23 where missing values were processed using a width=0.6 and shift=1.8). A two-sample t test between defined groups, using Benjamini and Hochberg correction to control the False Discovery Rate (FDR), was applied. Differentially expressed proteins were determined based on a q-value<0.05 and absolute value log2FC≥1. For the Gene Set Enrichment Analysis (GSEA) (22), we have used Cluster Profiler’s gseGO command with a p-value cutoff of 0.05 (Benjamini and Hochberg correction method), for significant “BP”, “MF” and “CC” terms of the GO analysis. R package Enrichplot (23) was used to visualize GSEA results. The results were represented using several visualization methods like, dot plot and gene-concept network (cnet plot) displaying linkages of genes and enriched terms.

Heatmaps were plotted using pheatmap R Package and ComplexHeatmap R package (24) for data validation. Principal component analysis (PCA) plot was used to visualize and interpret inter- and intragroup variability. PCA plot was made in Perseus 2.0.6.0 using log2 transformed LFQ intensity values after filtering the data for 100% valid values. The dataset was first subset for cell lines cultured under normal condition.

## 3 Results

### 3.1 Comparative proteomics study of pigmented and amelanotic metastatic melanoma cell lines

To understand the different metastatic melanoma cell behavior, we have used mass-spectrometry (MS) - based proteomics to profile existing differences between amelanotic and pigmented melanoma cells.

**Figure 1:**
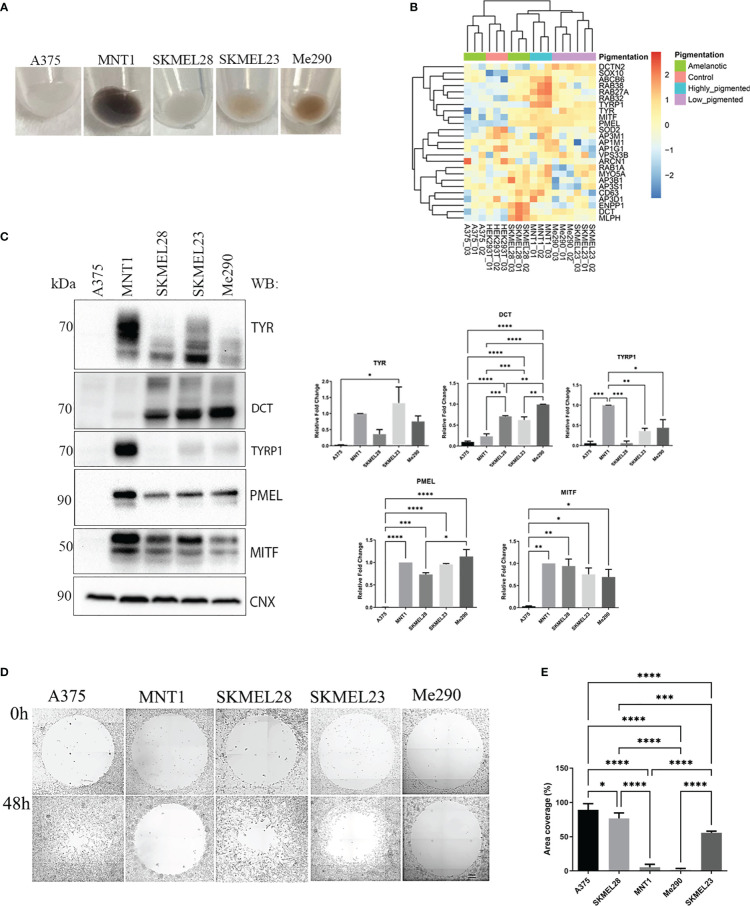
Expression of melanin related proteins in pigmented and amelanotic melanoma cells and assessment of cells migration. **(A)** Visual comparison between amelanotic cells (A375, SKMEL28) and pigmented cells (MNT1, SKMEL23, Me290). **(B)** Heatmap representation of log2 transformed LFQ intensity values for proteins annotated for pigmentation (GO:0043473). **(C)** Western blot analysis of TYR, DCT, TYRP1, PMEL, microphthalmia-associated transcription factor (MITF) protein expressions in A375, MNT1, SKMEL28, SKMEL23 and Me290 and quantitative representation of relative protein expressions (one-way ANOVA analysis; ****p < 0.0001; ***p < 0.001; **p < 0.01; *p < 0.05; n=3). Data are presented as mean ± SEM **(D)** Migration assay for A375, MNT1, SKMEL28, SKMEL23 and Me290 melanoma cell lines over a period of 2 days. **(E)** Measurements of area coverage indicating the significant differences between the five cell lines (one-way ANOVA analysis; ****p < 0.0001; ***p < 0.001). The measurements were conducted on at least 3 replicates.

Protein levels vary between cell lines which might be related to cell aggressiveness. By underlying these changes, we could understand where their characteristics come from. Nevertheless, the most notable aspect which we focused on was linked to those proteins that may be involved in migration and invasion. Five metastatic melanoma cell lines which differ in their pigmentation presence or absence were analyzed (**Figure 1A**). All five melanoma cell lines have some similar characteristics, originating from lymph nodes. Three of them, A375, SKMEL28 and MNT1 carry BRAF mutation, being at first sight very much alike, except for the pigmentation state, MNT1 being the only highly pigmentated cell line. As seen in **Figure 1A**, two cell lines, A375 and SKMEL28, were amelanotic and the other two, SKMEL23 and Me290 were low pigmented. We further analyzed the differences between non-pigmented A375, SKMEL28 cells and pigmented MNT1 cells. The proteomic analysis provided important differences at proteome level that stand for melanoma cell type differentiation and for the pigmentation process (**Figure 1B**, **Supplementary Tables 1, S1**). One of the most evident up-regulated melanoma specific transcription factors expressed in all melanoma cell lines was SOX10, while several proteins related to melanogenesis were increased only in MITF positive cell lines. Thus, RAB38, RAB27A, TYRP1 and ABCB6 were among the most abundant proteins in highly-pigmented MNT1 cells, compared to the other cell lines. On the contrary, the MITF negative A375 cell line, was more comparable with HEK293T, displaying less proteins involved in processes that may influence cell pigmentation (**Figure 1B**). Western blotting analysis confirmed the presence of pigmentation related proteins (TYR, dopachrome tautomerase (DCT), TYRP1, PMEL and MITF) in MNT1, SKMEL28, SKMEL23 and Me290 (**Figure 1C**). As expected, pigmentation varied mostly with the expression of MITF and TYR, but melanin synthesis and deposition was further modulated by PMEL, DCT and TYRP1, as previously suggested (25–27).

Next, we performed a migration assay in order to compare cell motility of pigmented (MNT1, SKMEL23 and Me290) versus nonpigmented (A375 and SKMEL28) melanoma cell lines and also for individual cell line migration capacity evaluation (**Figure 1D**). A375 and SKMEL28 displayed faster migration, when comparing with MNT1 and Me290 cells (**Figure 1E**). Overall, our migration test results confirmed the assumption according to which melanotic cell lines display a lower migration strength, A375 having the fastest migration capacity of all five metastatic cancer cell lines (28). A375 loss of pigmentation could suggest impairment in pigmentation process that occurs during melanoma progression. In the case of SKMEL28, its migration strength was higher than that of MNT1 and despite being a MITF positive cell line, lack of pigmentation is due to presence of immature stage I and II melanosomes (29). This difference may come from genetic alterations occurring in the melanoma cell. Loss of pigmentation may be one of the complex processes involved in melanoma cell shift from a less to a more aggressive phenotype. Although SKMEL28 cells displayed a large set of pigmentation-related proteins, lack of pigmentation may come from this transformation which arise during tumorigenesis. From the observed differences we could infer that there are coexisting changes at proteome level regarding cancer cell transformation in preference to tumor progression.

**Figure 2:**
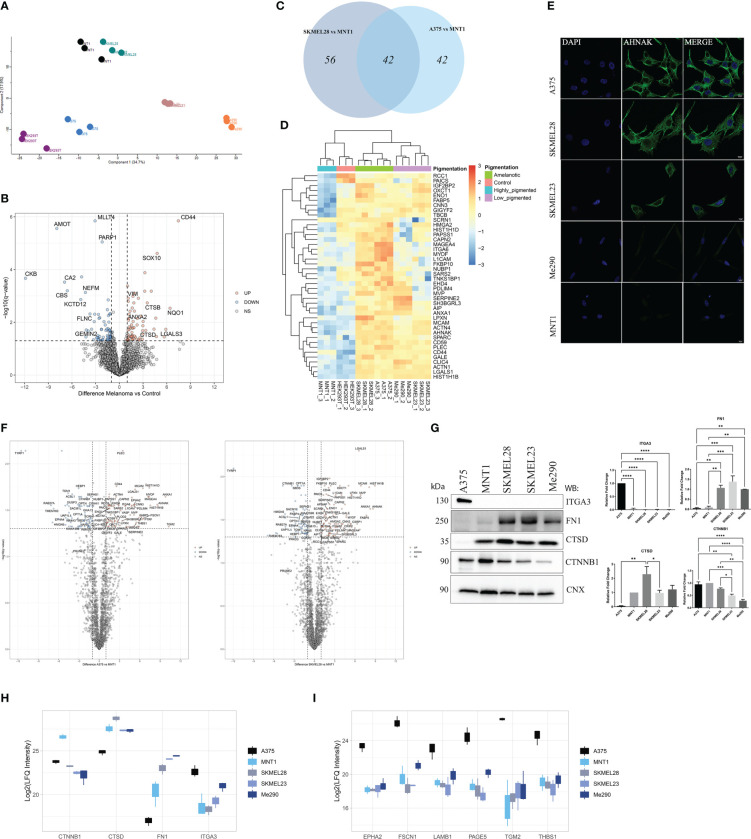
LC-MS/MS proteome comparison of amelanotic versus pigmented melanoma cells. **(A)** Principal Component Analysis (PCA) showing clustering of samples according to their features. **(B)** Volcano plot of protein expression differences (log2FC) vs -log10(q value) from two sample t test of melanoma samples vs control (q-value<0.05 and absolute log2FC≥1) (**Supplementary Table 1, S1**). **(C)** Venn diagram depicting shared upregulated proteins from the pairwise comparisons between A375 vs MNT1 and SKMEL28 vs MNT1. **(D)** Heatmap of log2 transformed LFQ intensity values showing the statistically significant shared up-regulated proteins from the comparisons between amelanotic cell lines (A375 and SKMEL28) and highly pigmented MNT1 cells (pairwise comparisons, two sample t test, q-value<0.05 and log2FC≥1) (**Supplementary Table 1, S1**). **(E)** Immunofluorescent staining of AHNAK in A375, SKMEL28, SKMEL23, Me290 and MNT1 cell lines. **(F)** Volcano plots highlighting differentially expressed proteins from the pairwise comparisons between amelanotic cells and highly pigmented MNT1 cells (q-value<0.05 and absolute log2FC≥1) (**Supplementary Table 1, S1**). **(G)** Western blotting assays and intensity band quantification for ITGA3, FN1, CTSD and CTNNB1 protein levels (one-way ANOVA analysis; ***p <0.001; **p<0.01; *p<0.05, n=3). Calnexin (CNX) was used as internal control. Data are presented as mean ± SEM. **(H)** Box plot of log2 transformed LFQ intensity values of CTNNB1, CTSD, FN1 and ITGA3 (**Supplementary Table 1, S1**). **(I)** Box plot of log2 transformed LFQ intensity values for EPHA2, FSCN1, LAMB1, PAGE5, TGM2 and THBS1 (**Supplementary Table 1, S1**).

In order to provide insight into differences that stand for variations in cell migration capacity, the proteomic data were correlated with migration assay results. Our proteomic approach involved using HEK293T as a negative control when identifying new potential melanoma biomarkers. Principal component analysis revealed samples clustering according to their similarity (**Figure 2A**). When comparing HEK293T cells to melanoma cells, we observed that among the significantly up-regulated proteins in HEK293T, there are proteins that might be tumor suppressor proteins, such as CKB that was low expressed in tumor cells (30) (**Figure 2B**). For instance, we have identified angiomotin protein (AMOT) as being one of the most strongly down-regulated protein in all five melanoma cell lines. Its high expression level in HEK293T cells might be correlated with absence of malignancy, considering its decreased level in melanoma cells.

Regarding proteins that might be predictable for fast-migration capacity or might rely on the amelanotic state of A375 and SKMEL28, 42 proteins were significantly up-regulated (q-value<0.05, log2FC≥1) in both amelanotic cell lines compared to MNT1 cells (**Figures 2C, D**). Considering that SKMEL28 and MNT1 cells share common characteristics as emerge from PCA plot, we can assume that SKMEL28 has an intermediate proteome profile between MNT1 cells and A375. This is also based on the presence to a certain extent of several pigmentation related proteins and the migration assay results. Hierarchical clustering of samples based on significantly up-regulated proteins in amelanotic cells compared to MNT1 cells showed that SKMEL23 and Me290 have an intermediate profile and selected proteins set apart these cells from the two extreme conditions. As shown above, in terms of cell motility, A375, SKMEL28, and SKMEL23 displayed increased migration capacity, as opposed to MNT1 and Me290. This property might reside in the similar significantly up-regulated proteins shared by these three cell lines, which includes neuroblast differentiation-associated protein AHNAK (AHNAK), melanoma cell adhesion molecule (MCAM) and high mobility group protein A2 (HMGA2). To confirm the proteomic data, low AHNAK protein level in MNT1 and Me290 cells was further validated by immunofluorescence assay (**Figure 2E**).

The two amelanotic cell lines (A375 and SKMEL28) seemed to be comparable not only by their migration strength, but also at the proteome level. Identified up-regulated proteins indicated a cancer specific pattern that help differentiate melanoma subtypes. ANXA1, AHNAK, MCAM, myoferlin (MYOF) and galectin-1(LGALS1) were among the most up-regulated proteins in amelanotic cells compared to highly pigmented MNT1, while catenin beta-1 (CTNNB1) was strongly up-regulated in MNT1 cells (**Figure 2F**). A greater attention should also be given to plectin (PLEC), another high molecular weight protein just as AHNAK, that seemed to be poorly expressed in MNT1 cells and whose elevated level was a major common feature of amelanotic cells. To the contrary, ephrin type-A receptor 4 (EPHA4) and protein PRUNE2 were up-regulated in MNT1 cells. Even though the difference for PRUNE2 expression between cell lines was not significant, its increased level might be defining for their low ability to migrate. EPHA4 role in overcoming malignant progression was already demonstrated (31) and this observation supported the proteomic data, EPHA4 being overexpressed in MNT1 slow-migrating cells (**Supplementary Table 1, S1**).

We have also underlined changes in cytoskeleton network of MNT1 cells, where alpha-actinin-1 (ACTN1) and alpha-actinin-4 (ACTN4) had a significantly decreased level. Also, ANXA1 might present melanoma cell type specificity and according to our data together with PLEC were strongly down-regulated in highly pigmented MNT1 cells.

Moreover, differences in protein level were noticed for CTNNB1, cathepsin D (CTSD), FN1 and integrin alpha-3 (ITGA3). The proteomics results were further validated by Western blot (**Figure 2G, H**). In addition, ephrin type-A receptor 2 (EPHA2), fascin actin-bundling protein 1 (FSCN1), laminin subunit beta 1 (LAMB1), P antigen family member 5 (PAGE5), transglutaminase 2 (TGM2) and thrombospondin (THBS1) displayed a cell type specific pattern that underlines melanoma heterogeneity, being up-regulated in A375 cells (**Figure 2I**).

**Figure 3:**
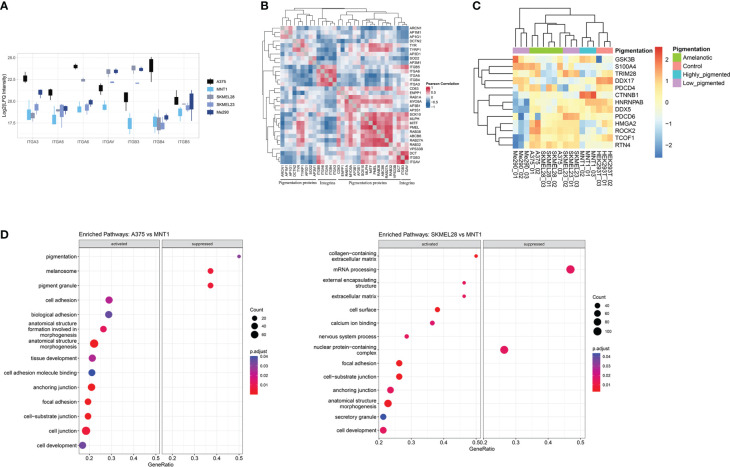
Proteins involved in cellular adhesion process are downregulated in highly pigmented melanoma cells. **(A)** Box plot of log2 transformed LFQ intensity of selected integrins showing the differences between cell lines and a prevalence of integrins to have an increased expression in A375 cell line. **(B)** Pearson’s protein-protein correlation matrix of integrins and proteins annotated for pigmentation (GO:0043473). **(C)** Heatmap of log2 transformed LFQ intensity values of proteins related to epithelial to mesenchymal transition (GO:0001837) identified in our proteomic data. This set of proteins differentiate low migrating cells (Me290 and MNT1) from the more aggressive counterparts, A375, SKMEL28 and SKMEL23. **(D)** Dot plots showing the results of GSEA analysis associated with “biological process”, “molecular function” and “cellular component” terms (p.adjust<0.05), coming from the pairwise comparison between amelanotic (A375 and SKMEL28) and highly pigmented cells (MNT1) (**Supplementary Table 1**, GSEA A375 vs MNT1 and GSEA SKMEL28 vs MNT1).

Furthermore, the decreased expression of pigmentation-related proteins together with the increased level of integrins can be defining for the A375 cells phenotype (**Figure 3A**). The expression of integrins could be viewed as being inversely correlated with the presence of melanogenetic proteins, which might confirm that pigmentation loss is evidence of a more aggressive phenotype (**Figure 3B**). Integrin alpha-6 (ITGA6) showed higher expression in amelanotic cells and therefore its increased expression can be associated with disease progression. Hence, our data demonstrated not only specific features at the cytoskeleton level, but also at integrin expression.

Further, we analysed different aspects related to increased cell migration capacity. A major determinant for enhanced cell motility may be due to a shift from epithelial to mesenchymal phenotype, defined as epithelial-mesenchymal transition (EMT) (**Figure 3C**). This process is correlated with cancer invasion, disease severity and is reflected in altered protein expression. During EMT, cells lose epithelial characteristics and gain mesenchymal traits, leading to enhanced migratory ability, by which cells reach distant tissue sites. Several EMT biomarkers were proposed, among which stand out cell-surface proteins, cytoskeletal proteins, transcription factors and extracellular matrix proteins (32). We succeeded in identifying common proteins related to EMT transition in the two amelanotic cancer cell lines (A375 and SKMEL28), which supports our hypothesis regarding amelanotic cell lines aggressiveness. Amelanotic cells, together with low-pigmented SKMEL23 that also displayed increased migration capacity, were linked by EMT-related proteins. All these findings are proof that amelanotic cell lines share common conventional features and also distinct characteristics regarding cell ability to spread. The observed differences are consistent with the possibility to subclassify metastatic melanoma cells relying on the identified proteins.

Gene set enrichment analysis underlines several biological processes that were found to be up-regulated in amelanotic cells, among which was defined “ focal adhesion” complex (**Figure 3D**, **Supplementary Table 1**, GSEA A375 vs MNT1 and GSEA SKMEL28 vs MNT1). The core enrichment genes related to “focal adhesion” complex consists of proteins like TGM2, LPXN, EPHA2 and ITGA6.

By grouping the samples according to their pigmentation state, the number of differentially expressed proteins increased (**Supplemetary Table 1, S2** and **Supplementary Figures 1A, B**). Most of the proposed biomarkers were validated by both approaches and are mainly represented by proteins with large differences in their expression level between cell lines, making them accessible candidates for melanoma subphenotyping (**Table 1**, **Supplemetary Table 1, S2**).

**Table 1:** Top 10 up-regulated proteins in amelanotic melanoma compared to highly pigmented MNT1 cells.

Accession No.	Gene names	Log2FC
P04083	ANXA1	9.02
Q09666	AHNAK	8.60
P16401	HIST1H1B	8.24
Q01469	FABP5	6.79
P43121	MCAM	6.65
P16402	HIST1H1D	6.44
P06703	S100A6	6.32
Q9NZM1-6	MYOF	5.99
Q14764	MVP	5.98
P09382	LGALS1	5.93

Log2FC= Student’s t test difference Amelanotic vs Highly pigmented.

Basically, our proposed set of proteins might represent potential diagnostic criteria for melanoma cell evaluation. Cell ability to spread and to develop distant metastases correlated with the lack of pigmentation, might be a determinant for tumor invasiveness. In MITF-positive metastatic melanoma cell lines, SKMEL28 and MNT1, the observed differences might arise from cell metastasis-initiating capacity. Relying on these differences previously demonstrated, we assume that also other mentioned proteins might be related to cancer cell migration. We can hypothesize that presence or expression over a certain limit of the above-mentioned proteins could help to differentiate an invasive cancer from a less aggressive one.

### 3.2 The influence of hypoxia on the melanoma cells proteome

**Figure 4:**
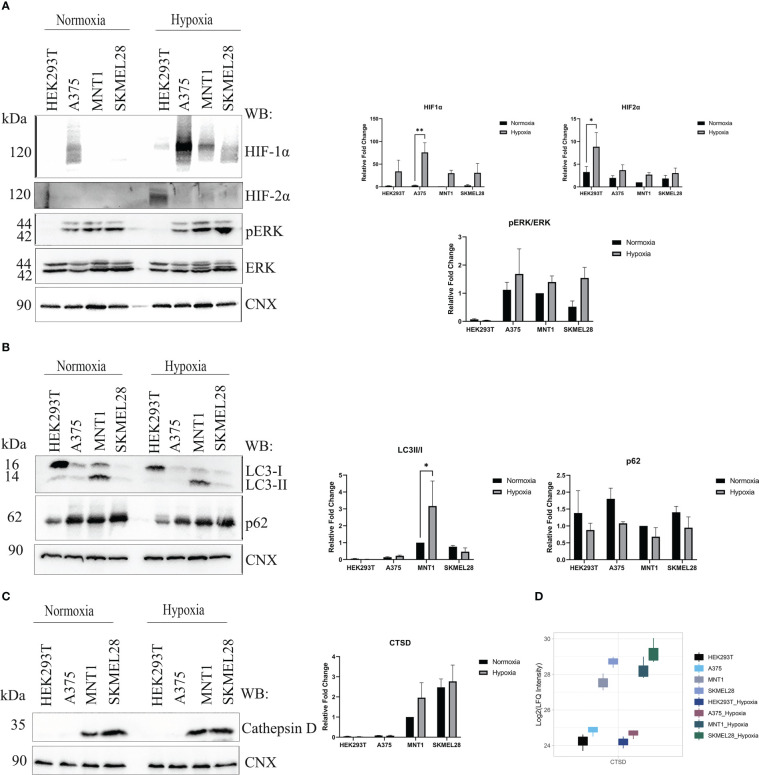
Hypoxia influence on several processes upon melanoma cells cultivation under low oxygen condition (1% O_2_, 5%CO_2_ and 94% N_2_). **(A)** Western blot assay of hypoxia-inducible factor 1-alpha (HIF1α), hypoxia-inducible factor 2-alpha (HIF2α), phosphorylated extracellular signal-regulated kinase (p-ERK) and extracellular signal-regulated kinases (ERK) protein expressions in HEK293T, A375, MNT1 and SKMEL28 cells. HEK293T, A375, MNT1 and SKMEL28 cells were kept in normoxia and hypoxia (1% O2) for 24 hours, at 37°C. Cells were harvested in RIPA buffer and specific proteins were identified. Expression levels of HIF1α and HIF2α proteins were normalized to calnexin (n=3). **(B)** Western blotting of autophagy related proteins (LC3 and p62). Quantitative analysis of LC3-II to LC3-I protein ratio, (n=3). **(C)** The same experiment as in **(A)** was performed for cathepsin D protein expression. Densitometric quantification of cathepsin D protein bands was assessed using calnexin as internal control. Data are represented as mean ± SEM (two-way ANOVA with Sidak multiple comparisons test; **p < 0.01, *p < 0.05). **(D)** Box plot of log2 transformed LFQ intensity values of cathepsin D in normoxia and hypoxia, as emerge from mass spectrometry data results.

To investigate the alterations of the gene expression in a micro-environment that accompany cancer progression, melanoma cells were exposed to low oxygen concentration (1% O2, 24 h) and proteomic changes within every cell line were analyzed. All three melanoma cell lines (A375, MNT1 and SKMEL28) performed well under hypoxic environment and several mechanisms that are partly responsible for cellular adaptation to hypoxia were highlighted. Presence of HIF1α and HIF2α validated the existence of a hypoxic environment and cells seemed to respond differently to changes in oxygen level (**Figure 4A**). A375 presented the highest HIF1α protein level both in hypoxia and normoxia compared to the other cell lines. The presence of a low level of HIF1α in normoxia in A375 may be due to a constitutive expression. Meanwhile, HIF2α was more abundant in HEK293T hypoxic cells, while HIF1α was poorly expressed. Thereby, hypoxic condition might exert a cell type specific response. HEK293T seemed to respond more rapidly to chronic exposure to hypoxia than melanoma cell lines, after 24 hours of cell culturing in presence of 1% O2. Further, the influence of hypoxic shift on MAPK/ERK pathway was studied. A modest increased level of phosphorylated ERK (pERK) was observed in A375, MNT1 and SKMEL28 hypoxic cells compared to those grown in normoxia.

Apart from ERK pathway activation, the role of autophagy in promoting cell survival was also studied. Variability of LC3-II/LC3-I ratio was noted in all four cell lines and decreased p62 level may be due to increased autophagic flux in hypoxia (**Figure 4B**). It is worth mentioning the increased LC3-II level in MNT1 cells which could indicate a constitutively active autophagy, that differentiates these cells from the others. Moreover, autophagy seemed to be highly regulated in MNT1 cells in presence of low oxygen level. Taken together, this data show that hypoxia modulates cell survival mechanism and their ability to acquire an invasive phenotype. We aimed to validate cathepsin D protein level by Western blot in normoxia and hypoxia and despite the fact that the results were not statistically significant, the Western blot results (**Figure 4C**) and the proteomic data (**Figure 4D**) indicate the highest cathepsin D level occurring in SKMEL28 and MNT1 cells. Being a marker of aggressiveness, cathepsin D expression was increased during cells exposure to hypoxia, especially in MNT1 and SKMEL28 cells, highlighting hypoxia role in promoting metastatic cell potential. Cathepsin D knock-down in zebrafish (33) was also associated with skin hyperpigmentation, that might suggests cathepsin D involvement in pigmentation changes during hypoxia exposure of cancer cell lines.

**Figure 5:**
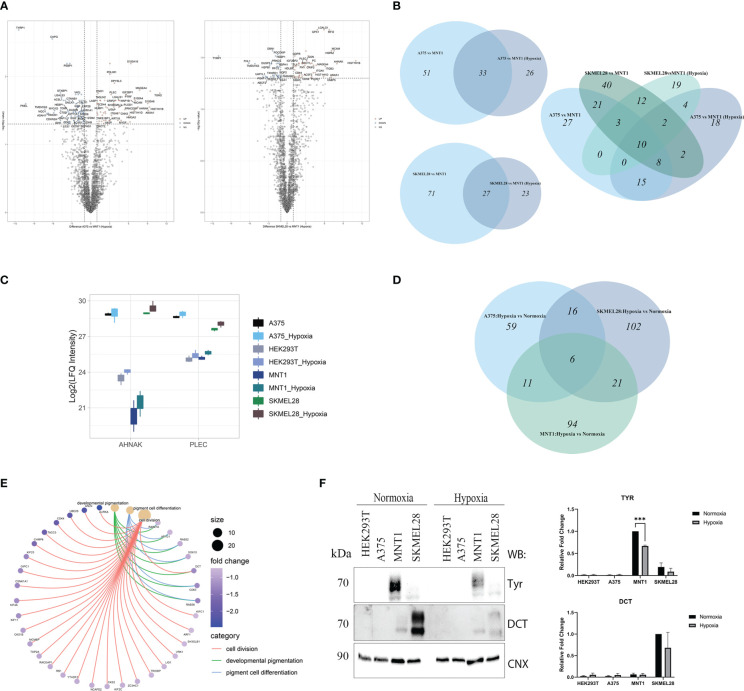
LC-MS/MS proteome analysis of melanoma cells in hypoxia. **(A)** Volcano plots representing the protein expression changes under hypoxia in the comparisons between amelanotic and highly pigmented cells (pairwise comparisons, q value<0.05, absolute log2FC ≥1) (**Supplementary Table 1, S1**). **(B)** Venn diagrams depicting unique and shared sets of up-regulated proteins from the comparisons between amelanotic and highly pigmented cells in normoxia and hypoxia **(C)** Box plot of log2 transformed LFQ intensity level of AHNAK and PLEC. **(D)** Venn diagram depicting unique and shared sets of up-regulated proteins in melanoma cell lines after cell exposure to hypoxia (log2FC≥1). **(E)** Cnet plot depicting the linkages between core enriched proteins for processes that were significantly enriched, coming from the comparison between melanotic cells cultured under hypoxia vs normoxia (**Supplementary Table 1**, GSEA MNT1 Hypoxia vs Normoxia). **(F)** Western blot analysis of TYR and DCT under normoxia and hypoxia. Calnexin was used as loading control. Western blot densitometry band quantification for TYR and DCT is presented as the mean ± SEM (two-way ANOVA with Sidak multiple comparisons test; ***p < 0.001, n=3).

The decreased number of significantly up-regulated proteins coming from the comparison between amelanotic cells with highly-pigmented MNT1 cell line under low oxygen level accentuated the proteomic shift that melanoma cells were experiencing (**Figures 5A, B**). Despite the low level of differentially expressed proteins, other 4 proteins came out being significantly up-regulated in amelanotic cells, namely HLA-DRB1, ME2, PLOD1, ANLN. A total of 10 proteins remained significantly up-regulated in A375 and SKMEL28 cells in both comparisons (normoxia and hypoxia): LGALS1, AHNAK, PLEC, CD44, ANXA1, MCAM, HIST1H1B, HIST1H1D, MAGEA4 and OXCT1. The number of significantly common upregulated proteins in amelanotic cells compared to MNT1 in normoxia and hypoxia increases when samples were grouped and compared according to their phenotype (**Supplementary Figures 1C, D**). 42 proteins were found to be significantly upregulated in amelanotic cells in both conditions and for instance, differences in protein level for ASPH between cell lines become more evident. The protein was further validated in public datasets as associated to amelanotic phenotype. A slightly elevated level of PLEC and AHNAK proteins in hypoxic cells can be associated with their presumable role in malignant progression (**Figure 5C**). The comparison between amelanotic cells and its highly pigmented counterparts under hypoxia indicates that CDK2 protein is also significantly downregulated in A375, as in normoxia in SKMEL28. Taking into account that we have compared metastatic melanoma cell lines with different migration strengths, increased CDK2 protein level in MNT1 confirms its importance as a prognosis biomarker. Its elevated level in metastatic lesions confirms lymph node infiltration, but in the same time its reduced expression is correlated with deep infiltration and poor prognosis (34). These observations are in concordance with our results that demonstrate CDK2 increased level in slow-migrating cells (MNT1) that originate from lymph nodes and its decreased protein expression in A375 and SKMEL28 cells.

No significant changes were observed in protein level under hypoxia. Nevertheless, by using a log2FC cutoff greater than 1, we managed to notice the resemblance of SKMEL28 with MNT1 cell lines, based on shared upregulated proteins (**Figure 5D**). Of all three melanoma cell lines, only MNT1 cells seemed to be subjected to complex changes at proteome level under hypoxia and gene set enrichment analysis revealed several processes that are impaired. The most important one was the “developmental pigmentation” process, which was down-regulated in hypoxic MNT1 cells due to global change in the level of proteins involved in pigmentation (**Figure 5E**, **Supplementary Table 1**, GSEA MNT1 Hypoxia vs Normoxia).

Hypoxia influence on pigmentation related proteins was further validated by Western blotting (**Figure 5F**). We considered DCT and TYR protein level measurements for showing the changes in protein abundance between conditions.

Regarding the expression level of the main panel of biomarkers that we have already identified in normoxia, AHNAK, MYOF, ANXA1, CAPN2, ACTN4 along with proteins involved in cell adhesion/migration (integrins, PLEC), the results indicate that the cultivation conditions do not interfere with the biomarkers abundance. Therefore, this algorithm could be used in assessing a tumor proteome irrespective of the tumor oxygen level known to be highly variable in different tissues.

### 3.3 Data validation using available public datasets

**Figure 6:**
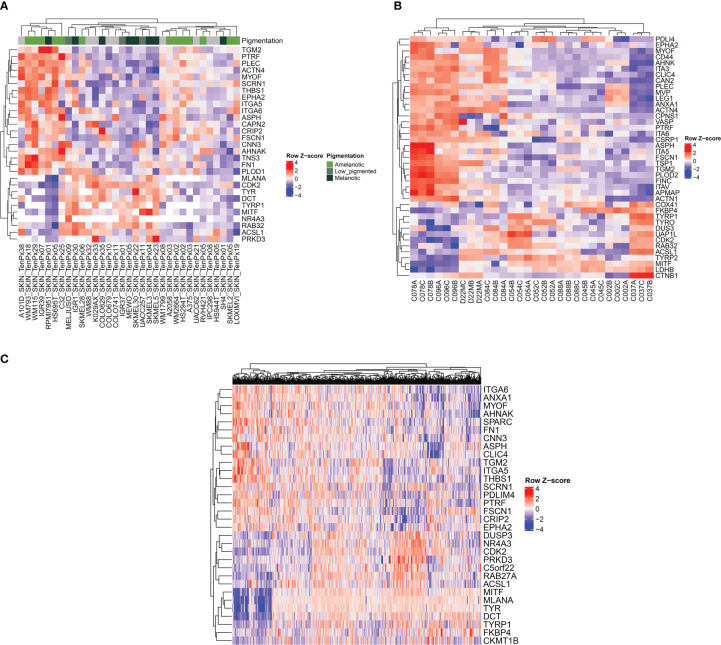
Heatmap representations showing the relationship between melanogenetic proteins and selected proteins identified in this study. The analysis was conducted on three different public datasets. **(A)** Heatmap of z-score of normalized protein expression values for selected proteins identified in 33 melanoma cell lines (52). **(B)** Heatmap of z-score of normalized protein area (log2) for selected proteins. Three biological replicates isolated from patients with melanoma are represented (53). **(C)** Heatmap of RNA-seq expression z-scores (RSEM z score) for selected protein-coding genes of 442 melanoma patients (54).

To validate the proteomic signatures obtained from the five melanoma cell lines analyzed, we used several public datasets including entries from cohorts of melanoma patients. For this analysis, we considered two main subtypes, the amelanotic and pigmented melanomas differing in the expression of the following genes: TYR, TYRP1, TYRP2, RAB32, RAB27A, MITF and MLANA rather than subtyping melanomas according to combined melanocytic and neural crest genes and MITF regulated genes (35, 36). First, we have analyzed data from 33 melanoma cell lines (37–51) provided by The Cancer Cell Line Encyclopedia (CCLE) proteomic dataset (52) (**Figure 6A**). We found three main clusters separated based on their pigmentation genes (**Figure 6A**, left and right clusters: - low expression of pigmentation genes and the middle cluster- expression of pigmentation genes). The most extreme conditions represented by high abundance of melanogenetic proteins and low expression levels of several proteins that we found differentially expressed were negatively correlated. Moreover, a similar pattern was observed for proteins that were significantly up-regulated in A375 (TGM2, FSCN1, THBS1 and EPHA2) or SKMEL28 (FN1) cells. In the low pigment clusters, 12 out of 19 cell lines were reported as amelanotic and in the high pigment cluster, 9 out of 14 cell lines were reported as pigmented (**Supplementary Table 2**). This confirms that the selected panel of proteins is specific for pigmentation in melanoma cells. Also, the signature of the amelanotic cells recapitulates the one proposed by us for the analysed amelanotic melanoma cells.

A second proteomics dataset published by Molloy’s laboratory was considered further for validation (53) (**Figure 6B**). The analyzed samples from ten different patients assembled in two main clusters depending on the abundance of the pigmentation genes, with subclusters that could reflect variable levels of the melanin synthesis genes. Indeed, the cell lines with high PMEL content displayed non-pigmented lysates, whilst all the low PMEL cells were pigmented, as reported by the author. As in the case of the first dataset, the expression of proteins which are part of the melanogenic regulatory pathway were inversely correlated with the main proteins displaying important differences between cell lines. Among them there are AHNAK, PLEC, integrin alpha-V (ITGA5), ITGA3, ITGA6, MYOF, CD44, ANXA1, CAPN2 and CSRP1. Interestingly, the authors made a correlation between MEKi resistance and the increased protein level of ITGAV, FSCN1 and CD44, that we have also found to be downregulated in MNT1 cells. In the same time they noticed that proteins responsible to melanogenesis process were inversely correlated with MEKi resistance. They reported that “C078”, “C084” and “C096” cell lines were the most resistant to therapy. Taken together their observations and our results that show “C078” and “C096” to display low pigmentation proteins and increased proposed biomarkers we can assume that amelanotic melanoma might be more aggressive than their counterparts, but further studies are required. Therefore, the panel of biomarkers which we are proposing could be of predictive value for melanoma evaluation.

Further, to assess the robustness of our predictions obtained based on proteomics data, we have analyzed a melanoma transcriptomic published dataset from Cancer Genome Atlas (TCGA-SKCM) (54). This dataset had the advantage of including a higher cohort, including 442 patients. The validation here was limited by the lack of clinical information regarding the pigmentation status of the tumors. However, applying the above algorithm, we found at transcriptional level a pattern consisting of several genes among which it is worth mentioning ANXA1, ITGA6, AHNAK, MYOF, FN1, ASPH, EPHA2, THBS1 that might be influenced by protein coding genes responsible for melanin biosynthesis pathway (**Figure 6C**). Due to the limited clinical data that were available, further validations are required to confirm the correlation between low pigmentation proteins and the proposed pattern. In addition, several proteins that were significantly up-regulated in MNT1 cells were also directly correlated with increased level of pigmentation-related proteins and are represented mainly by ACSL1, DUSP3, PRKD3 and FKBP4. We propose that there might be a specific signature for melanotic cells as well.

All these results show that the panel of biomarkers found experimentally by the analysis of five melanoma cell lines with different melanin content, could be successfully validated in two public datasets including 43 proteomic samples, correlating pigmentation with a number of genes whose expression is down-regulated in melanoma patients.

## 4 Discussion

Among cancers, melanoma is unique in that it produces melanin pigments. Involving more than 125 genes, pigmentation not only confers a distinctive feature, but mobilizes important proteomic resources within the melanoma cell (55). Disruptions of the function of these genes lead to hypopigmented or completely amelanotic melanomas that are tumorigenic and highly metastatic (4, 5) Whilst amelanotic melanoma has been found to be deadlier by most studies, there are controversial results about the surviving days period when compared with pigmented lesions, mostly due to the advanced stages diagnostic of these tumors (3–5, 56–59). In a recent survey involving 342 patients with amelanotic melanoma, it has been found that 25% were clinically and 12% pathologically misdiagnosed (60).Overall proteome differences between amelanotic and melanotic cells can represent a starting point in understanding features that could be linked to aggressiveness of the cells. Analyzing two amelanotic and three melanotic cell lines with different degree of pigmentation, we found several proteins that were down-regulated in the pigmented cells MNT1, Me290 and SKMEL23. The hyperpigmented MNT1 cells were previously found to have a similar profile to normal melanocytes and were also demonstrated to be nontumorigenic in mice (61, 62). By assigning a low migration capacity to MNT1 cells and taking into consideration their pigmentation which differentiate them from A375 and SKMEL28, we identified a series of target proteins that could be related to cell migration. Importantly, the identified proteins were partially confirmed for other two melanotic cell lines, i.e. Me290 and SKMEL23, both less pigmented than MNT1 cells. All analysed pigmented cells express a higher level of MITF than the A375 amelanotic cell. Our observation also highlights the need of a more complex set of pigmentation related proteins to classify melanoma according to its pigmentation status, given that only by MITF we could not discriminate the amelanotic phenotype of SKMEL28 from the other three pigmented cell lines included in the study. This transcription factor that promotes the expression of the most melanogenic proteins including TYR, TYRP1, DCT and PMEL, was found to be also critical for melanoma invasion and proliferation upon down-regulation in melanoma cells (28). Another protein that is significantly reduced in amelanotic cells is CDK2, one of the regulators of cell cycle progression (63). Both MITF and CDK2 could influence the expression of a number of genes associated with melanoma invasion, whilst being dynamically regulated by other genes within the melanoma cell (64). Hence, despite their vital roles within the cell, the dynamic regulation of these two proteins could lead to both an invasive or to a non-invasive phenotype, making the role of the other regulated proteins equally significant.

One of the most important proteins that has been found to be poorly expressed in MNT1 cells is AHNAK. Its involvement in cell migration and invasion could represent a significant sign of poor prognosis with associated metastases. Its mechanism of action involves the activation of TGFβ signaling pathway which subsequently leads to epithelial-mesenchymal transition (EMT) (65). Moreover, AHNAK overexpression predicts poor clinical outcome in larynx carcinoma patients (66) and reduced actin cytoskeleton dynamics and suppression of cell migration was assessed recently by AHNAK knock-down in six metastatic tumor cell lines (67). Other cytoskeletal and extracellular proteins such as PLEC, ACTN4, and LGALS1 can be regarded as promising indicators of disease progression. PLEC expression was recently found to be up-regulated in metastatic growth phase melanoma and it seems to be differently expressed in melanoma than in normal skin tissue (68). PLEC is abundant in the two most metastatic cell lines, being very low in MNT1, which may indicate that the intermediate filament may not be linked in the focal adhesion points of MNT1 in the presence of low level of PLEC. This, in turn, could affect the actin migration in the EMT of MNT1 cells (69–71). Increased differences between pigmented and non-pigmented cell lines were also noted for calpain 2 (CAPN2), aspartate beta-hydroxylase (ASPH) and MYOF. Another family of proteins that are adhesion molecules and receptors of extracellular matrix, integrins, were found up-regulated in the amelanotic cells. Cell spreading and acquiring of an invasive character are consequences of the presence of high number of other similar proteins to those mentioned above.

Hypoxia influences cell phenotype, highlighted by loss of several pigmentation-related proteins. Importantly, the panel of specific biomarkers with high expression in normoxia in amelanotic melanoma cells remained unambiguously high during hypoxia, indicating the robustness of the discovered proteomic pattern in various tumor conditions and validating their specificity for melanoma diagnostic.

Considering the above data, we selected a panel of biomarkers that are systematically detected by the LC-MS/MS analyses even when low amounts of sample are available, that we propose as a diagnostic tool for melanoma evaluation. The panel consist of several proteins that shows an increased expression in amelanotic melanoma cells compared to pigmented cells, of which the most important ones seem to be represented by AHNAK, PLEC, FN1, ANXA1, ASPH, MYOF, THBS1, EPHA2, ITGA6, ITGA5, FSCN1, TGM2, ACTN4 and CAPN2. Their inverse correlation with the abundance of proteins involved in pigmentation was validated in at least two datasets.

To validate the prognostic value of this set of proteins, we analyzed their frequency in reported proteomic datasets produced by different laboratories. A limited validation of the results came from the analysis of the *TCGA* database (54) that allowed us to perform a similar analysis on 442 patients with melanoma at a transcriptomic level, in the absence of clinical data related to the pigmentation of the tumors. The above panel of biomarkers clustered into a low expression profile of the selected biomarkers in melanomas with pigment genes and a different cluster with up-regulated biomarkers. The analysis of a public proteomics dataset, The Cancer Cell Line Encyclopedia (CCLE) displaying data of 33 melanoma cell lines showed grouping of samples with high pigmentation proteins and low expression of 19 proteins that include CRIP2, FSCN1, PTRF, PLOD1, ACTN4, ITGA5, ITGA6, CNN3, FN1, THBS1, EPHA2, SCRN1, CAPN2, ASPH, PLEC, AHNAK, TGM2, MYOF, TNS3 and a different cluster including melanoma cell lines with low expression of melanin genes and an increased level of the above mentioned proteins (**Figure 6A**). Next, we examined the data reported recently by the Molloy laboratory (53), that analyzed samples coming from patients with metastatic BRAF/NRAS melanoma. We have compared the quantitative proteomes of melanoma patients with high and low expression of the melanogenic proteins TYR, TYRP1, PMEL, MITF, CDK2, and found that most of the predicted markers, including AHNAK, PLEC, ANXA1, integrins, MYOF identified patients with a protein pattern that might define amelanotic melanoma. The proteomic signature of the two types of melanoma patients recapitulates our panel of biomarkers proposed to discriminate between the aggressive low pigmented/amelanotic melanoma on one side and pigmented melanoma on the other side. Therefore, the fact that similar signatures as the one reported in this work occur in the proteomic pattern of a cohort of melanoma patients, further supports the new biomarkers profile proposed here.

## 5 Conclusions

Low migration capacity of hyper-pigmented cells was demonstrated and correlated with several low expressed proteins related to cell invasion and migration. According to our study, there are significant differences at proteome level between pigmented and amelanotic melanoma cell lines. We thus enlarge the panel of proteins that differentiate pigmented and nonpigmented melanomas with new markers that can be identified and relatively quantified by mass spectrometry. These differences can be associated with both pigmentation and cell migration capacity and selected proteins might be viewed as promising prognostic biomarker candidates for disease progression and also for distant metastasis susceptibility.

## Data availability statement

The datasets presented in this study can be found in online repositories (72). The names of the repository/repositories and accession number(s) can be found below: https://www.ebi.ac.uk/pride/archive/, PXD033192.

## Author contributions

Conceptualization, SMP; methodology, SMP and IVM; validation, IVM, AAR and GM; formal analysis, IVM; investigation, IVM, AAR and GM; data curation, IVM; raw mass-spectrometry analysis CVM; writing - original draft preparation, investigation, SMP and IVM; writing - review and editing, SMP; supervision, SMP; project administration, SMP; All authors have read and agreed to the published version of the manuscript. All authors contributed to the article and approved the submitted version.

## Supplementary material

Supplementary Figure 1 LC-MS/MS proteome comparison of melanoma cells grouped according to their phenotype **(A)** Volcano plot showing the log2FC vs -log10(q value) from two sample t test of amelanotic cells vs highly pigmented MNT1 cell line. **(B)** Heatmap of log2 transformed LFQ intensity values showing the statistically significant up-regulated proteins from the comparison between amelanotic cell lines and highly pigmented MNT1 cells. **(C)** Venn diagram depicting unique and shared set of upregulated proteins in amelanotic cells vs MNT1 cells in both comparisons (normoxia and hypoxia). **(D)** Heatmap of significantly upregulated proteins in amelanotic cells compared to highly-pigmented MNT1 cells in hypoxia (q value<0.05 and log2FC≥1)
