# Supplementary figures and images for: New panel of biomarkers to discriminate between amelanotic and melanotic metastatic melanoma
Source: Front Oncol. 2023 Jan 26;12:1061832. doi: 10.3389/fonc.2022.1061832 (PMC9909407; doi:10.3389/fonc.2022.1061832)

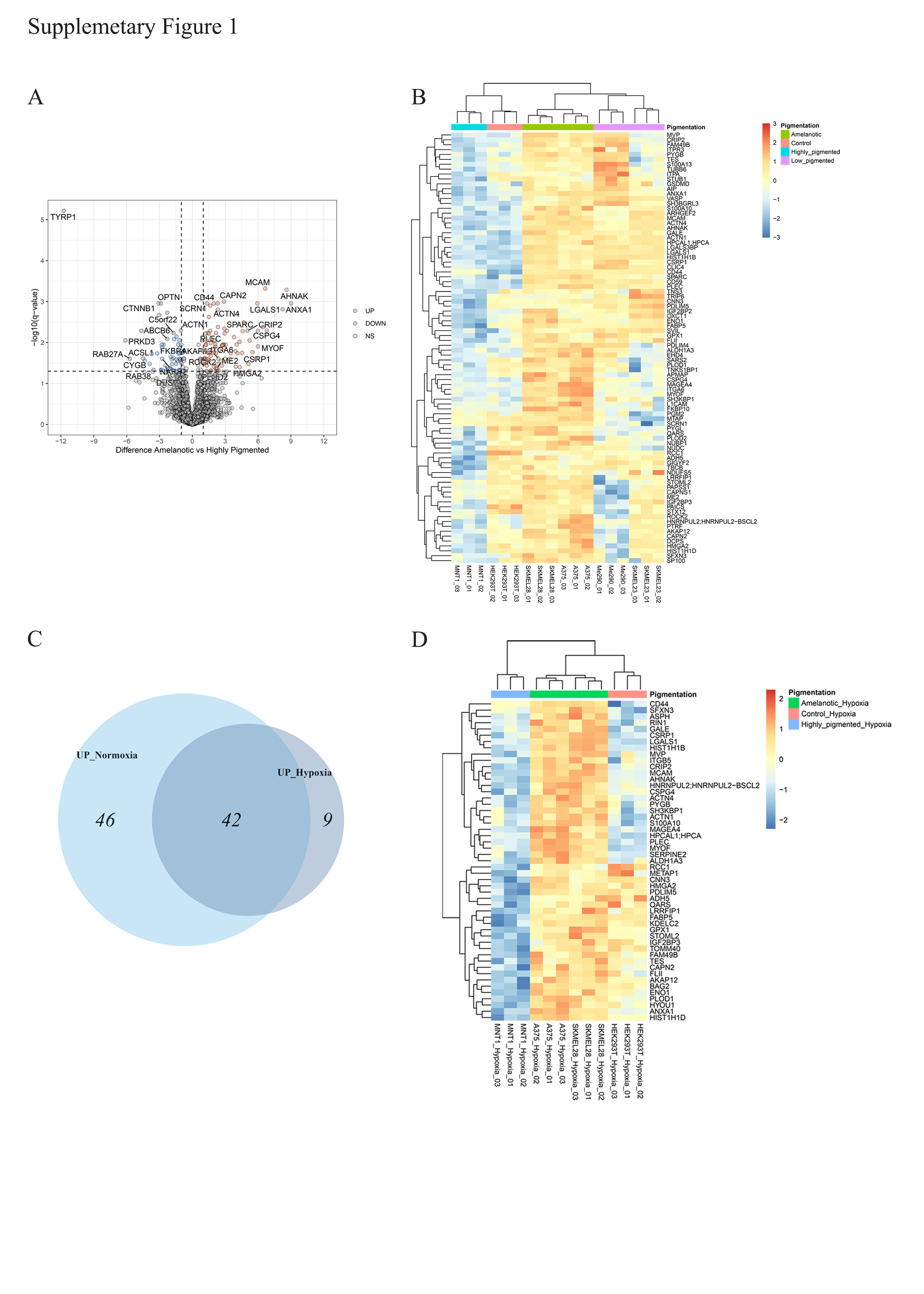

Supplement: Supplementary Figure 1 — LC-MS/MS proteome comparison of melanoma cells grouped according to their phenotype (A) Volcano plot showing the log2FC vs -log10(q value) from two sample t test of amelanotic cells vs highly pigmented MNT1 cell line. (B) Heatmap of log2 transformed LFQ intensity values showing the statistically significant up-regulated proteins from the comparison between amelanotic cell lines and highly pigmented MNT1 cells. (C) Venn diagram depicting unique and shared set of upregulated proteins in amelanotic cells vs MNT1 cells in both comparisons (normoxia and hypoxia). (D) Heatmap of significantly upregulated proteins in amelanotic cells compared to highly-pigmented MNT1 cells in hypoxia (q value<0.05 and log2FC≥1) [file Image_1.tif]
