# Supplementary material for: New panel of biomarkers to discriminate between amelanotic and melanotic metastatic melanoma
Source: Front Oncol. 2023 Jan 26;12:1061832. doi: 10.3389/fonc.2022.1061832 (PMC9909407; doi:10.3389/fonc.2022.1061832)
Supplement: Supplementary file 3 [file Table_2.docx]

| Cell line | Pigmentation | Reference |
| --- | --- | --- |
| A101D | Skin | ATCC |
| LOXIMVI | Amelanotic | (37) |
| SKMEL2 | Amelanotic | (38) |
| HS944T | * |  |
| HS294T | Amelanotic | (39) |
| A375 | Amelanotic | (37) |
| A2058 | Amelanotic | (37) |
| UACC62 | * |  |
| RVH421 | Amelanotic | (40) |
| IGR39 | Amelanotic | (41) |
| HS695T | Amelanotic | ATCC |
| RPMI7951 | Melanotic | ATCC |
| WM793 | Amelanotic | (42) |
| WM115 | Amelanotic | (43) |
| MEWO | Melanotic | (38) |
| IGR37 | Melanotic (weak) | (41) |
| SH4 | Melanotic | (44) |
| COLO741 | * |  |
| COLO679 | Skin | *Library of Integrated Network-based Cellular Signatures* |
| IPC298 | * |  |
| WM2664 | Amelanotic | (38) |
| C32 | Amelanotic | (37) |
| WM1799 | * |  |
| K029AX | Skin | (45) |
| COLO829 | Melanotic | (46) |
| SKMEL30 | Melanotic | (37) |
| SKMEL5 | Melanotic | (38) |
| WM88 | Skin | (45) |
| SKMEL28 | Amelanotic | (47) |
| MELJUSO | Low pigmented | (48) |
| IGR1 | Melanotic | (49) |
| SKMEL3 | Melanotic | (50) |
| UACC257 | Slightly pigmented | (51) |

*Information not found
